# Modifiable Risk Factors for Cardiovascular Disease in Children with Type 1 Diabetes: Can Early Intervention Prevent Future Cardiovascular Events?

**DOI:** 10.1007/s11892-017-0968-y

**Published:** 2017-11-03

**Authors:** Evgenia Gourgari, Dana Dabelea, Kristina Rother

**Affiliations:** 1 Department of Pediatrics, Georgetown University, Washington DC, USA; 2 Section on Endocrinology and Genetics, Program on Developmental Endocrinology & Genetics (PDEGEN) and Pediatric Endocrinology Inter-Institute Training Program, Eunice Kennedy Shriver National Institute of Child Health and Human Development (NICHD), Bethesda, MD USA; 3 Department of Epidemiology, Colorado School of Public Health, University of Colorado Denver, Aurora, CO USA; 4 Section on Pediatric Diabetes and Metabolism, National Institute of Diabetes and Digestive and Kidney Diseases (NIDDK), National Institutes of Health, Bethesda, MD USA

**Keywords:** Type 1 diabetes, Children, Youth, Cardiovascular risk, Intervention

## Abstract

**Purpose of Review:**

Patients with type 1 diabetes have increased risk for cardiovascular disease.

The purpose of this review is to examine the following:

i) current evidence for subclinical cardiovascular disease (CVD) in children with type 1 diabetes (T1DM)
ii) known modifiable risk factors for CVD and their relationship to subclinical CVD in this population
iii) studies that have addressed these risk factors in order to improve CVD outcomes in children with T1DM

**Recent Findings:**

Subclinical CVD presents in children as increased carotid intima-media thickness, increased arterial stiffness, and endothelial and myocardial dysfunction. Modifiable risk factors for CVD include hyperglycemia, hyperlipidemia, obesity, hypertension, depression, and autonomic dysfunction. Very few randomized controlled studies have been done in children with T1DM to examine how modification of these risk factors can affect their CVD.

**Summary:**

Children with T1DM have subclinical CVD and multiple modifiable risk factors for CVD. More research is needed to define how modification of these factors affects the progression of CVD.

## Introduction

Type 1 diabetes (T1DM) affects approximately 3 million people in the USA of whom approximately 200,000 are children [1]. The prevalence of T1DM among pediatric patients has increased between 2001 and 2009 in the USA and worldwide [2, 3]. Children with T1DM are diagnosed as early as the first year of life, and by the time they are young adults, most of them have been potentially exposed to chronic hyperglycemia for more than two decades. By the time they reach 55 years of age, 35% of patients with T1D will die from CVD compared to only 8% of non-diabetic men and 4% of women [4]. This increased risk for CVD starts in childhood, and it is estimated that 14–45% of children with T1DM have more than two CVD risk factors [5••]. Furthermore, children with T1DM already have evidence of subclinical CVD, which can present as increased arterial stiffness, carotid intima-media thickness, and endothelial dysfunction. While most interventions to decrease CVD have been studied in adults, few clinical trials have investigated the safety and efficacy of preventive measurements in pediatric patients.

The purpose of this review is to describe:

i) current evidence for subclinical CVD in children with T1DM
ii) known modifiable risk factors for CVD and their relationship to subclinical CVD in this population and
iii) studies that have addressed these risk factors in order to improve CVD outcomes for children with T1DM

## Methods

We searched PubMed for articles in English using the keywords “type 1 diabetes,” “cardiovascular disease,” “children,” “microalbuminuria,” “arterial stiffness,” and “depression” as well as “statin,” “metformin,” “ACE inhibitor,” “non-insulin therapies,” “lifestyle,” and “intervention.” We also reviewed manuscripts found in the references of the articles we found via the PubMed search.

## Manifestations of Subclinical Cardiovascular Disease

Major cardiovascular events, such as myocardial infarction and stroke, do not manifest at a young age, and thus, investigators use indirect methods to measure subclinical CVD. The most commonly used methods to detect subclinical CVD are described below.

### Carotid Intima-Media Thickness

One of the methods to investigate CVD is the use of carotid intima-media thickness (CIMT), which has been shown in adults to correlate with future cardiovascular events [6]. Results from studies of CIMT in children with T1DM have been conflicting in some cases, perhaps due to different patient characteristics, sample size and methods used to measure CIMT, but the majority of studies have shown higher CIMT in children with T1DM vs those without diabetes [7, 8]. An older study found higher CIMT in 85 children with T1DM with an average age of 11 ± 2 years, HbA1c 8.9 ± 1.4%, and duration of diabetes of 4.4 ± 3.3 years compared to healthy controls (HC) (0.47 ± 0.04 versus 0.42 ± 0.04 mm, *P* < 0.0001) [8]. Another study also found CIMT to be higher in 52 children with T1DM with an average age of 11.8 ± 3.1 years, HbA1c of 8.6 ± 1.6%, and duration of diabetes of 4.8 ± 3.2 years compared to 47 HC (0.463 ± 0.04 versus 0.441 ± 0.04 mm, *P* = 0.001) [9]. In another study, 142 patients with T1DM with an average age of 16 ± 2.6 years had higher CIMT when compared to 87 HC (0.564 ± 0.005 versus 0.540 ± 0.006 mm, *P* = 0.002) [10]. The SEARCH for Diabetes in Youth (SEARCH) study was initiated in 2000 and is funded from the Centers for Disease Control and Prevention and the National Institute of Diabetes and Digestive and Kidney Diseases, in five sites across the USA to improve understanding of childhood diabetes and has recruited only youth with diabetes. SEARCH CVD is an ancillary study conducted at two sites in the USA that collects data on subclinical CVD in youth with diabetes and healthy controls 10–25 years old. The SEARCH for Diabetes in Youth Case-Control (SEARCH CC) is another ancillary study conducted at two sites in the USA that collected clinical and metabolic data on youth with diabetes and healthy controls 10–22 years old. In SEARCH CVD, 402 T1DM patients with an average age of 18.8 ± 3.3 years, HbA1c 8.9 ± 1.8%, and duration of diabetes 9.8 ± 3.8 years, no difference was seen in the common or internal CIMT when compared to 206 HC (0.449 ± 0.073 versus 0.450 ± 0.067 mm, *P* = 0.82 and 0.399 ± 0.078 versus 0.396 ± 0.073 mm, *P* = 0.68, respectively), but higher CIMT was found at the level of the bulb of the carotid artery in the T1DM group compared to HC (0.461 ± 0.073 versus 0.445 ± 0.069 mm, *P* = 0.01) [7]. Another study showed no difference in CIMT (0.43 ± 0.05 versus 0.42 ± 0.05, *P* = 0.35) but showed higher aortic intima-media thickness (0.57 ± 0.11 versus 0.50 ± 0.07, *P* < 0.001) in 66 children with T1DM of average age 14.1 ± 2.5 years when compared to HC [11]. More recent studies have also shown higher CIMT in children with T1DM compared to HC [12–14].

### Arterial Stiffness

Another approach to evaluate subclinical CVD is the arterial stiffness. Studies in adults have shown that higher arterial stiffness is associated with increased risk for cardiovascular events [15, 16]. The carotid-femoral pulse wave velocity (PWV) is considered the gold standard to measure arterial stiffness [17]. Another variable called augmentation index @ 75 beats per minute (AI75) is also used, although AI75 is influenced by peripheral arterial stiffness, heart rate, and reflected waves from peripheral arteries arriving at the heart during systole [17]. Haller et al. reported that 98 children with T1DM with an average HbA1c of 8.41 ± 1.29% had higher AI75 compared to 57 HC matched for age, sex, race, and BMI [18]. In the SEARCH study, 535 youths with T1DM with an average age of 14.6 ± 3.3 years and HbA1c 8.3 ± 1.9% had higher arterial stiffness when compared to 241 healthy controls [19]. Later, the same group reported in the SEARCH CVD study 402 adolescents and young adults with T1DM with an average age of 18.8 ± 3.3 years, HbA1c 8.9 ± 1.8%, and duration of T1DM 9.8 ± 3.8 years had higher pulse wave velocity and AI75 compared to 206 healthy controls in a cross-sectional study [20]. Another study found higher PWV among 72 children with T1DM with an average age of 12.3 ± 1.6, HbA1c 8.6 ± 1.9%, and diabetes duration of 3.9 ± 2.6 years when compared to 77 HC. In a different study that used brachial-ankle pulse wave velocity as an index of arterial stiffness, no difference was seen among 87 children with T1DM with median HbA1c 9.1% and duration of diabetes 5.1 years compared to 21 HC [21]. Similar results were also seen in a recent study with 199 youths with T1DM compared to 178 HC [22].

### Endothelial Dysfunction

Endothelial dysfunction (ED) is another surrogate marker of subclinical CVD which has been linked to increased CVD events and can be measured by flow-mediated dilation, usually of the brachial artery [23]. Multiple studies have shown worse ED in children with T1DM [24–31], and we will only describe a few of them. A study found greater ED measured by flow-mediated dilation (FMD) among 21 pre-adolescent children with T1DM with an average age of 8.3 ± 0.3 years old, HbA1c of 8.0 ± 2.0%, and diabetes duration of 4.3 ± 0.4 years when compared to 15 age matched HC (7.1 ± 0.8% versus 9.8 ± 1.1%, *P* = 0.04) [32]. Another study showed similar results with 23 children with T1DM with an average age of 14.6 ± 1.75 years, HbA1c 8.3 ± 1.6%, and diabetes duration of 5.8 ± 3.6 years when compared to 23 HC measuring the ED with peripheral artery tonometry at the distal phalanges of the hand (1.78 ± 0.4 versus 2.06 ± 0.4, *P* = 0.02) [25]. A more recent study also showed more ED measured with FMD among 30 children with T1DM with an average age of 11.1 ± 3.8 years, HbA1c 9.7 ± 2.2%, and duration of diabetes 3.9 ± 0.6 years when compared to 30 HC (4.60 ± 2.13% versus 9.31 ± 2.29%, *P* < 0.001) [26].

### Myocardial Dysfunction

Investigation of myocardial function using echocardiography has also shown increased subclinical CVD in children with T1DM with impaired diastolic dysfunction and left ventricular hypertrophy when compared to HC [22, 26, 33–35]. Diastolic dysfunction was worst in T1DM adolescents compared to HC (early peak velocity/atrial filling velocity (E/A) ratio 1.45 ± 0.19 versus 1.85 ± 0.34, *P* < 0.001) [26]. Adolescents with T1DM have smaller left ventricle end-systolic dimension (2.90 ± 0.32 versus 2.95 ± 0.33, *P* = 0.0032) and other left ventricular myocardial deformation indices [22].

In summary, the above studies indicate that children with T1DM have evidence of subclinical CVD even at this very young age. The main risk factors that contribute to high CVD risk in children with T1DM are described below.

## Risk Factors for Cardiovascular Disease

### Hyperglycemia

Data from the Diabetes Control and Complications Trial (DCCT) that has the longest follow-up (30 years) of adult patients with T1DM have shown that hyperglycemia as assessed by mean HbA1c correlated significantly with the longitudinal changes in all of the traditional CVD risk factors [36]. In fact, the strongest longitudinal associations were among time-dependent triglycerides and LDL Cholesterol (LDL-C) (β-estimates 5.86 ± 0.39, *P* < 0.0001 and 2.65 ± 0.23, *P* < 0.0001) with time-dependent HbA1c [36]. More importantly, a recent study using multivariate models assessed the association of traditional and novel risk factors—such as hypertension, hyperglycemia defined by average HbA1c, duration of diabetes, nephropathy, hypoglycemic events, hyperlipidemia, family history of diabetes, and smoking—with major atherosclerotic cardiovascular events in the DCCT cohort and found that HbA1c is an extremely important risk factor for major cardiovascular events, and less important only to age [37••].

In pediatric studies, we have seen similar results from cross-sectional and retrospective studies, indirectly assessing the effect of hyperglycemia on established CVD risk factors such as the LDL-C. In the SEARCH study, there were significantly higher levels of total cholesterol, LDL-C, triglycerides, and non-HDL-C with higher hemoglobin A1c among 1680 children with T1DM, but no association was seen between glycemia and HDL-C [38]. Poor glycemic control was associated with high non-HDL and total cholesterol among 682 children with T1DM seen at Barbara Davis Center for Childhood Diabetes [39]. In another retrospective cohort study of 572 youths with T1DM followed longitudinally for a median of 9.3 years, HbA1c modestly affected LDL-C and non-HDL cholesterol [40].

In the SEARCH CVD study, adjustment for HbA1c eliminated the differences in CIMT between T1DM and HC, suggesting that the differences in CIMT are attributable to poor glycemic control [7]. A correlation of CIMT and HbA1c has also been found in other studies [9, 41].

In SEARCH CVD, 298 youths with T1DM with an average age 14.5 years and duration of diabetes 4.8 years had two measurements of arterial stiffness conducted 5 years apart [42]. Authors found that worsening glycemic control was associated with increased arterial stiffness over time [42]. In a cross-sectional evaluation of arterial stiffness among 535 subjects with T1DM and 241 HC, the presence of T1DM status was associated with higher arterial stiffness although the HbA1c was not included in the regression model in this manuscript [19].

The relationship of poor glycemia and endothelial dysfunction, high circulating endothelial cells—which are markers of endothelial cell damage—was correlated with HbA1c in the study by Eltayeb et al. [26]. Another recent study looked at the endothelial dysfunction assessed with skin microvascular perfusion in 181 youths with T1DM and found that HbA1c was an independent determinant of microvascular perfusion [28].

### Hyperlipidemia

Previous studies have shown that approximately 15% of children with T1DM have high cholesterol, in particular LDL Cholesterol, which is a well-established risk factor for CVD [39, 43, 44 **••**]. In another study published by the SEARCH for Diabetes in Youth Case-Control (SEARCH CC) study, 512 children with T1DM with an average duration of diabetes of 4.2 years had higher small dense LDL particles (*P* < 0.001 for children with HbA1C ≥ 7.5%) and higher Apo-lipoprotein B (ApoB) (*P* < 0.0001) compared to 188 healthy controls [43]. ApoB is a marker of the sum of atherogenic lipoproteins (LDL cholesterol and VLDL cholesterol) because all atherogenic lipoproteins carry the ApoB molecule [45]. Interestingly, the small dense LDL cholesterol and the Apo-B were higher in T1DM patients irrespective of their glycemic control, indicating they could be used as better markers for stratifying CVD risk in this population [43]. A recent study investigated further the role of apolipoprotein-B in CVD risk in 267 adolescents with T1DM who had at least 5 years duration of diabetes and an average HbA1c 8.9 ± 1.6%. They found that patients with high Apo-B had significantly higher arterial stiffness (measured by PWV), which again suggests that the addition of Apo-B can be a useful tool for stratifying the CVD risk in children with T1DM, potentially better than the traditional lipid panel [45]. In the study by Rodriguez et al., LDL-C was independently associated with CIMT [9].

### Obesity, Insulin Resistance, and Lack of Exercise

Contrary to the typical clinical picture of a thin patient with T1DM, obesity is very common today in children with T1DM. Based on baseline data in the T1D Exchange registry obtained from 5529 adolescents with T1D with an average age of 15.4 ± 1.4 years and HbA1c 8.7 ± 1.8%, overweight status was present in 22.9% and obesity was present in 13.1% in the overall sample [46]. Obesity was highest among girls (40.8%) and adolescents of Hispanic/Latino race/ethnicity (46.1%) [46]. Lower frequency was seen in the European German/Austrian DPV multicenter survey: 12.5% of T1DM patients were overweight and 2.8% were obese among 12,774 patients that were followed prospectively with a mean age of 13.4 ± 3.9 and mean diabetes duration of 4.7 ± 3.0 years [47]. In the European study, factors that were associated with increased obesity during the course of diabetes were higher insulin doses, female gender, low BMI at diabetes onset, intensified insulin therapy, pubertal diabetes onset, and long duration of diabetes [47]. When data from the two registries were combined, children with T1DM had higher BMI *z* score than the international and their respective normal national data, indicating that obesity among children with T1DM is a significant problem [48]. This is in accordance with adult data from the DCCT trial, where patients with T1DM who received the higher insulin doses also had increased weight gain and higher total cholesterol and LDL-C, central obesity, insulin resistance, blood pressure, more coronary artery calcifications, and higher CIMT on follow-up, underlying the role of obesity in promoting CVD in patients with T1DM [49].

Obesity is associated with atherogenic lipoproteins in youth with T1DM, which increases their CVD risk [39, 50, 51], and BMI is associated with worsening LDL-C over time [40]. In the SEARCH CVD study, 298 youths with T1DM and an average age of 13.3 ± 2.9 years, who had two study visits 5 years apart, BMI was the only modifiable risk factor that predicted CIMT based on a linear regression model [52]. Obesity is associated with arterial stiffness even in children without diabetes [53], and it is not surprising that adiposity was also found to be independently correlated to increased arterial stiffness in the previously mentioned SEARCH CVD study by Shah et al. [20].

Insulin resistance (IR) or low insulin sensitivity is associated with obesity and metabolic syndrome. While traditionally IR (or low insulin sensitivity) has been thought to be part of the pathophysiology of type 2 diabetes, recent evidence suggests that IR is also a feature of T1DM that is present even in lean T1DM, and it is a risk factor for CVD in T1DM [44 **••**, 54, 55]. In fact, studies in adults have shown that patients with T1DM and high IR have more coronary artery calcifications, suggesting a higher CVD risk [56]. Recent studies have also shown that IR can also be present in children with T1DM who have normal BMI, suggesting a different underlying mechanism for IR in T1DM [33]. A recent study showed that children with T1DM have atherogenic lipid profiles with dense LDL-C and HDL-cholesterol that is associated with low insulin sensitivity [57]. The same group by Nadeau et al. also showed that children with T1DM and low insulin sensitivity had impaired functional exercise capacity and that low insulin sensitivity correlated with peak oxygen consumption, a measurement of cardiopulmonary fitness [33]. Another large study investigated the effect of low IS on cardiovascular risk factors among 292 adolescents with T1DM with an average age of 15.4 ± 2.1 years, hemoglobin A1c 8.9 ± 1.6%, and duration of diabetes 8.8 ± 3.0 years compared to 89 HC [58]. The authors found that lower IS was associated with worsening CVD risk factors of blood pressure, fasting total, low-density lipoprotein-cholesterol (LDL-C) and high-density lipoprotein-cholesterol (HDL-C), high sensitivity C-reactive protein, and BMI *z* score [58]. Low insulin sensitivity was also found to be an important risk factor for increased arterial stiffness over time in youth with T1DM in the SEARCH CVD study [59].

The American Diabetes Association recommends 60 min of daily exercise for youth with diabetes; however, only 8% achieve that goal [60, 61]. Youth with T1DM tends to exercise less than youth without diabetes, most likely due to fear of hypoglycemia due to insulin [44 **••**, 60]. Studies have shown that youth with T1DM who exercises more have better HbA1c, lipoprotein profile, and blood pressure [60, 62] and can therefore improve their CVD risk. A recent study showed that children with T1DM who were making less than 10,000 steps per day had worst arterial intima-media thickness compared to their peers who were more physically active [63].

### Hypertension

Hypertension is an established risk factor for CVD. The American Diabetes Association recommends a goal of BP that is below the 90th percentile for age and sex in children with T1DM and approximately 4–7% of children with T1DM have hypertension [44 **••**]. Studies have shown that even though the blood pressure in the office can be normal, children with T1DM can have high nighttime blood pressure and lack the physiologic nocturnal dip in blood pressure, which can be measured with 24 h ambulatory blood pressure monitor (ABPM) [44 **••**].

Hypertension in children with T1DM in the SEARCH CVD study has been linked to arterial stiffness and CIMT [7, 20, 42]. In a recent study, 52 children and adolescents with an average age of 14.07 ± 3.03 years and duration of diabetes 5.13 ± 2.18 years had higher ABPM readings compared to 20 HC and within the study group subjects with an abnormal ABPM reading had significantly higher concentrations of sE-selectin—a marker of endothelial dysfunction—compared with subjects with normal ABPM [29]. Another large study evaluated 24-h ABPM in 159 children with T1DM compared to 100 HC and found that 23.3% of children with T1DM had hypertension, which is more frequent than what is usually reported as hypertension using office measurements of blood pressure, suggesting that ABPM might be a better tool to detect hypertension at the early stages [41]. Furthermore, authors found that the loss of nocturnal systolic blood pressure dip was a significant predictor of CIMT—along with HbA1c—as previously described by another group [41, 64].

### Microalbuminuria

Microalbuminuria is a marker of diffuse vascular injury but in both adults and adolescents with T1DM, can be a self-limiting reversible condition that often resolves with no intervention or with improvement of glycemic control [5, 65–67]. Studies in adults with T1DM have shown patients with T1DM, and microalbuminuria has increased CVD and mortality risk; however, this may represent the fact that microalbuminuria can be mediated by the presence of other CVD risk factors such as hypertension, hyperlipidemia, and IR [5]. Microalbuminuria can also progress to macroalbuminuria and diabetic kidney disease, which is also an established risk factor of CVD in adults with T1DM [5]. In the T1D Exchange, approximately 4.4% of the 7549 youths with T1DM had microalbuminuria which was associated with longer duration of diabetes, higher HbA1c, older age, female sex, higher diastolic blood pressure (BP), and lower BMI [68]. Another pediatric study showed that 406 adolescents with T1DM with an average age of 14.1 ± 1.9 years and diabetes duration of 6.7 ± 3.7 years had evidence of early atherosclerosis based on aortic intima-media thickness when compared to 57 HC and that higher urinary albumin excretion, even within the normal range, was associated with early atherosclerosis in youth with T1DM [69].

### Depression

Depression is common among pediatric patients with T1DM with one large study reporting depressive symptoms in 17% of patients with T1DM [70]. Depression can play a detrimental role in the adherence to diabetes therapy and in the blood glucose monitoring and indirectly affect CVD risk by worsening hyperglycemia and subsequently lipoprotein profile as well [44 **••**, 71, 72]. The American Diabetes Association recommends to consider screening for depression in children as young as 7 or 8 years old [61].

### Autonomic Dysfunction

While autonomic dysfunction is a sign of diabetic neuropathy, it has a direct effect on cardiovascular health for patients with T1DM because it affects the autonomic function of the heart. Autonomic dysfunction can present with low heart rate variability (HRV), which is associated with a 32–45% increased risk of cardiovascular events in subjects without established CVD [73]. Youth with T1DM from the SEARCH CVD study had decreased HRV which correlated with increased arterial stiffness [74–76]. Poor glycemic control defined as HbA1c over time appears to be a major determinant of decreased HRV [74, 77].

## Preventive Clinical Trials in Youth with T1DM

### Intensive Glycemic Control

Unfortunately, most youths with T1DM do not meet the ADA guidelines for adequate glycemic control and fail to achieve a glycemic control of < 7.5% [78]. It is concerning that among adolescents less than 21% achieve this target [78]. We know from the long-term follow-up of the Diabetes Control and Complications Trial (DCCT) cohort of adults with T1DM that improved hyperglycemia can improve significantly the cardiovascular outcomes and that glycemic control is closely correlated with other cardiovascular risk factors [36, 79]. All the interventions described above improve glycemic control in youth with T1DM; however, it remains a big challenge to implement these interventions in children. Collaborative efforts between health care professionals (pediatric endocrinologists, pediatricians, social workers, dietitians, diabetes educators, psychologists) and family members are needed to overcome the obstacles.

### Statin Medications

The American Diabetes Association recommends a statin medication in children with T1DM and LDL-C > 160 or > 130 mg/dl and additional cardiovascular risk factors [61]. This recommendation is based on studies done mainly in adults with T2DM and a few trials in adults with T1DM showing that statins can reduce CVD events in patients with diabetes [61, 80]. Very few randomized studies have been done in children with T1DM to investigate the effect of statin medications on CVD outcomes. One randomized, double-blinded, cross-over pilot study of 12 weeks using 20 mg atorvastatin vs placebo showed no improvement on arterial stiffness and endothelial function on primary analysis, but a trend towards improvement in endothelial dysfunction was seen (*P* = 0.06) on a secondary proof of concept analysis [81]. In addition, the authors showed that there was a good efficacy profile with a reduction of 29 ± 20 mg/dl after 12 weeks of treatment, and the medication was well tolerated on the short term, without major adverse effects on AST or CPK and only a mild increase on ALT by 4.3 U/l [81]. Another randomized double-blinded study randomized 42 children with T1DM with average age 15 ± 0.3 years, HbA1c 8.8 ± 0.2%, and diabetes duration 6.8 ± 0.5 years to atorvastatin 20 mg daily or placebo for 6 months [82]. Patients were enrolled if they had LDL-C > 100 mg/dl and BMI < 95th percentile and excluded in they had LDL-C > 160 mg/dl [82]. Patients in the atorvastatin group had a baseline HbA1c that was significantly lower than the HbA1c of the placebo group but otherwise were not different in terms of their age, sex, race, and Tanner stage. The authors showed again that atorvastatin was overall safe and well-tolerated and one subject developed elevated creatinine kinase, which normalized after discontinuation of the medication [82]. They also reported a significant improvement on LDL-C, total LDL particles, non-HDL particles, and Apo-B. Another effort to initiate a randomized trial using statins in adolescents with T1DM indicated that poor recruitment of eligible youth with T1DM can be a major challenge in the conduct of these trials and collaborative efforts in multicenter trials are needed [83]. Another possible limitation to recruitment is the potential effect of teratogenicity of statins, which may necessitate the need for oral contraceptives in some female adolescents at high risk to become pregnant while using statin medications.

While all the above studies indicate improvement in lipid profile, there have been no long-term pediatric clinical trials to our knowledge investigating the effect of statin medications on measurements of subclinical CVD. One such trial is on-going and is expected to provide some evidence on this topic in the near future [84].

### Insulin Sensitizing and Incretin Mimetic Medications

Given the emerging role of insulin resistance in CVD risk in youth with T1DM, metformin—which improves insulin resistance—has been used as adjuvant treatment off label by pediatric endocrinologists mainly with the goal of improving glycemia. Recent randomized controlled pediatric studies have failed to demonstrate a significant benefit of metformin in improving glycemic control [85, 86]. A large multicenter double-blind, placebo-controlled randomized clinical trial enrolled 140 overweight/obese adolescents with an average 15.3 ± 1.7 years and diabetes duration 7.0 ± 3.3 years and treated them with metformin or placebo for 26 weeks [85]. The authors failed to demonstrate improvements in HbA1c or in the LDL-C, HDL, or total cholesterol; however, metformin decreased total daily dose and measurements of adiposity, in accordance with other similar studies [85, 87].

A systematic review and meta-analysis examined data from 325 patients with T1DM treated with metformin as adjuvant to insulin therapy [86] .The authors concluded that metformin has no benefit in improving glycemic control but it can lead to modest reduction in the total daily dose of insulin and the BMI, which suggests that metformin could be helpful in decreasing weight gain associated with insulin use and indirectly improve cardiovascular risk of youth with T1DM [86]. Another study was a 9-month randomized, double-blind, placebo controlled trial of metformin and placebo in 28 children with T1DM [88]. The authors showed no improvements in glycemic control or even in total daily dose; however, they state this could be due to the smaller size of the study [88]. An ongoing clinical trial aims to investigate the effect of metformin directly on CVD measurements such as flow-mediated dilation and carotid and aortic intima-media thickness and will be very informative [89].

Other incretin mimetic medications such as the GLP-1 agonists (exenatide, liraglutide) have been used in adults with T1DM and have shown some potential benefit in terms of improving their cardiovascular health [90]. Exenatide has been found to improve weight, insulin sensitivity, and lipoprotein particles in adults with T1DM [91]. Liraglutide use in adults with T1DM leads to reductions in their hypoglycemic events, total daily insulin dose, and bodyweight [90]. Very limited studies on GLP-1 agonists have been done in youth with T1DM. One small trial using exenatide in eight children with T1DM showed decreased total insulin dose and improved post-prandial hyperglycemia; however, the role of exenatide on CVD risk factors was not investigated [92].

Overall, more randomized controlled pediatric trials investigating the CVD effects of insulin sensitizing medications in youth with T1DM are needed [93].

### Antihypertensive Medications

ACE inhibitors and ARBs are the first line treatment options for persistent microalbuminuria and hypertension in youth with T1DM [5 **••**]. In a large database in Netherlands, ACE inhibitors, along with statins, are the most commonly used cardiovascular medications among youth with T1DM [94]. The T1D Exchange trial showed that while 4.4% of youth with T1DM have microalbuminuria, only 36% of them are treated with ACE inhibitors [68]. One potential explanation for this could be that microalbuminuria is often transient and some physicians elect the “wait and watch” approach over aggressive treatment. Another barrier is also the teratogenicity of both of these medications that can be problematic for certain adolescents with risky behaviors. Studies in adults with T1DM have shown no benefit in preventing diabetic kidney disease without the evidence of albuminuria or hypertension [61]. Based on previous pediatric studies, we know that ACE-inhibitors are effective in reducing persistent micro- and macroalbuminuria [67]; however, how this translates to direct benefit for CVD outcomes in children with T1DM is not known. Results from the ongoing trial AdTID will provide evidence on the impact of ACE inhibitors on CVD outcomes in youth with T1DM [84].

### Lifestyle Changes

American diabetes association recommends lifestyle change as the first line treatment to address high cholesterol, obesity, and hypertension in youth with diabetes [61]. A few randomized trials have looked at the effect of diet and exercise on CVD risk reduction in youth with T1DM. A 6-month prospective cohort trial aimed to promote Mediterranean style diet in 96 adolescents with T1DM using dietary intervention. The authors showed significant improvements with reduction of LDL cholesterol, non-HDL-cholesterol, and total cholesterol: HDL-cholesterol ratios in the intervention group (*P* < 0.001) [95]. Another study from the SEARCH study investigated the CVD effects of the DASH diet which encourages fruits, vegetables, low-fat milk products, whole grains, fish/poultry/nuts, lean red meats, and limited intake of sugar and sweets [96]. Authors found that better DASH diet score was significantly associated with lower HbA1c levels in youth with type 1 diabetes (*β*= − 0.20, *P* = 0.0063) and improved blood pressure in youth with T2DM (*β*= − 2.02, *P* = 0.0406) [96].

There have been some pediatric clinical trials looking at the effects of exercise on CVD health in youth with T1DM. In the large European database with 23,251 pediatric patients with T1DM, more exercise was associated with better cardiovascular risk profile, such as lower levels of LDL cholesterol (*P* < 0.005) and triglycerides (*P* < 0.00001) and higher levels of HDL cholesterol (*P* < 0.00001) in girls with T1DM while in boys there were differences only for triglycerides (*P* < 0.0005) and HDL cholesterol (*P* < 0.00001). Also, the percentage of patients with elevated diastolic blood pressure was lower in the exercise group (*P* < 0.005) and A1c was lower in the group of patients that exercised more (*P* < 0.000001) [62]. In an interventional trial, 15 children with T1DM received exercise training three times a week for 12 weeks [97]. After the intervention, the authors found significant improvements in total cholesterol levels (179.9 ± 2.6 versus 164.4 ± 11.4 mg/dl, *P* < 0.01) and in some parameters of the autonomic dysfunction (LF power and VLF power) [97]. In another study, seven children with T1DM received 18 weeks exercise training program, and at the end of the study, authors found improvements in physical fitness (measured by peak oxygen uptake during running test_,_ *P* = 0.039) and brachial artery endothelial dysfunction (measured by FMD, *P* < 0.05) [98]. In another trial, 16 subjects with T1DM were randomized to a twice per week combined aerobic and resistance training or regular physical activities for 20 weeks [99]. Authors found that exercise seemed to lower daily insulin requirements (0.96 versus 0.90 IU/kg/day, *P* < 0.05) and improved physical fitness (*P* < 0.05) [99]. A systematic review and meta-analysis of the exercise intervention trials in youth with T1DM analyzed data from 23 studies [100]. Authors concluded that exercise intervention has beneficial effects on HbA1c, BMI, triglycerides, and the total cholesterol [100]. The above studies indicate that exercise can be effective in improving CVD health in youth with T1DM.

### Antidepressant Medications

Depression is associated with poor glycemic control, and treatment with antidepressants is expected to improve control. A 6-month intervention trial with SSRI antidepressants was done in 58 adult patients with insulin-dependent poorly controlled T2DM [101]. That study showed that patients improved their depression and glycemic control and their HbA1c dropped from 8.5 ± 1.2 to 7.7 ± 0.7% (*P* < 0.001) without major changes in their lipid profile [101]. Other studies have shown deterioration of cardiovascular risk factors in adults treated with antidepressants and suggest aggressive monitoring of CVD risk in diabetic patients treated with antidepressants [102]. Based on the data from the observational German diabetes database, youth with T1DM and depression who were treated with antidepressants had better HbA1c compared to non-treated depressed youth (71.1 versus 78.1 mmol/l, *P* < 0.003) and a trend towards higher BMI (*P* = 0.05) [103]. To our knowledge, a randomized controlled study investigating the effects of antidepressants on CVD risk factors in youth with T1DM has not been yet conducted.

## Conclusions

Children with T1DM have evidence of subclinical CVD early in their life as evidenced by abnormalities in CIMT, arterial stiffness, endothelial dysfunction, and myocardial function. Multiple modifiable risk factors contribute to their increased risk for CVD, such as dyslipidemia, hyperglycemia, obesity, insulin resistance, lack of exercise, depression, and autonomic dysfunction. Limited pediatric randomized trials have been conducted to investigate the effect of improvement of these factors on subclinical CVD, but the results of the available trials are so far encouraging. More research is needed on this topic because addressing modifiable risk factors early in the course of CVD can lead to improved CVD outcomes and decrease future morbidity and mortality.
